# Exploring Web-Based Support for Suicidal Ideation in the Scottish Population: Usability Study

**DOI:** 10.2196/55932

**Published:** 2025-01-24

**Authors:** Heather McClelland, Rory C O'Connor, Laura Gibson, Donald J MacIntyre

**Affiliations:** 1 School of Health and Wellbeing University of Glasgow Glasgow United Kingdom; 2 Police Scotland Glasgow United Kingdom; 3 NHS Lothian Edinburgh United Kingdom

**Keywords:** suicide prevention, Scotland, suicidal thoughts, digital intervention, internet, self-help, crisis intervention

## Abstract

**Background:**

Suicide is a global health concern. In the United Kingdom, Scotland has the highest suicide rate. Lived experience and suicide prevention stakeholders in Scotland have identified a key gap in suicide prevention activities: the lack of 24-hour peer-driven web-based support for people who are suicidal.

**Objective:**

This usability study aimed to evaluate the feasibility, acceptability, utility, and reach of a suicide prevention website (Surviving Suicidal Thoughts) specifically designed to support residents in Scotland who are experiencing suicidal thoughts themselves or suspect or know someone who is experiencing suicidal thoughts. Intended support was delivered through the provision of personal testimony videos of individuals with lived experience.

**Methods:**

A peer-driven website was developed specifically to support residents of Scotland experiencing suicidal thoughts. The website included resources (eg, videos from lived experience and written guidance about how to respond to someone who may be experiencing suicidal thoughts) to help reduce distress, normalize experiences, and challenge distressing thoughts. The website was promoted via leading web-based social media channels and Google Ads. Evaluation of the website was based on website engagement, marketing strategy, and direct web user feedback via a cross-sectional survey.

**Results:**

Data were collected for 41 weeks (June 2022 to February 2023) spanning the launch of the website and the conclusion of the second marketing campaign. On average, the website received 99.9 visitors per day. A total of 56% (n=14,439) of visitors were female, ages ranged from younger than 18 years to older than 70 years (commonly between 25 and 34 years) and originated from all regions of Scotland. According to Google Search terms of Scottish residents, of the individuals indicated to be experiencing suicidal thoughts but not looking for help, 5.3% (n=920) engaged with the website compared to 10.5% (n=2898) who were indicated to be looking for help for themselves. Based on participant responses to the evaluation survey (n=101), the website was associated with a significant reduction in suicidal thoughts (*P*=.03). Reasons for visiting the website varied. Marketing data implied that people were more likely to engage with advertisements, which they felt were more personal, and visitors to the website were more likely to engage with videos, which corresponded to their age.

**Conclusions:**

A peer-led website may help residents of Scotland who are experiencing suicidal thoughts. Web-based interventions may have considerable reach in Scotland both in terms of age and geographic area. Engagement with the website was similar to other self-help websites for suicidal ideation; however, more nuanced methods of analyzing website engagement for help-seeking behavior are recommended. Future work would benefit from exploring the effectiveness of this website based on a larger participant sample with website modifications guided by the principles of social learning theory.

## Introduction

### Background

Suicide is a leading global health concern. The World Health Organization [1] estimates that 1 person dies by suicide every 40 seconds. In the United Kingdom, Scotland continues to have the highest suicide rate across its 4 nations [2-4]. Most people with thoughts of suicide do not seek professional help, and for those who do, too often they struggle to access timely care [5]. Therefore, improving timely and easy-to-access care for those experiencing suicidal ideation is a public health priority in Scotland.

In 2022, the Scottish government published a 10-year, suicide prevention strategy that aims to reduce suicide on a national level [6]. The strategy drew on evidence from over 200 Scottish suicide prevention stakeholders (eg, lived experience, mental health professionals, and charity organization professionals) [7], which indicated that digital peer support was among the most sought-after resource needed. Subsequently, the current strategy includes the following goal: “anyone in suicidal crisis should be able to access support remotely by phone, text or online” [6,7].

National strategies for suicide prevention are underpinned by the drive to understand human behavior within a psychosocial context [8]. The integrated motivational-volitional model of suicidal behavior [9,10] is a leading theory that maps the emergence of suicidal thoughts and behaviors. Specifically, it posits that feelings of defeat (in response to stress) can develop into entrapment, which, in the absence of factors such as social support and belongingness, can give rise to suicidal ideation and potentially suicidal behaviors.

However, other theories of human behavior can also be applied to understand suicide risk. Social learning theory [11], for example, posits that people are more likely to model their behavior on those with whom they identify [12]. Indeed, videos of individuals describing their own experience of, and surviving, thoughts of suicide have been shown to be effective in improving the life satisfaction of those who are experiencing suicidal thoughts themselves [13]. What is more, according to group identity theory [14], identifying with others with shared thoughts or experiences can facilitate feelings of belongingness, the absence of which is consistently associated with suicide risk [13,14].

Systematic reviews by both Lai et al [15] and Robinson et al [16] indicate that a range of web-based suicide prevention strategies (including self-guided, digital cognitive behavioral therapy, web chat, and signposting to resources) are effective in reducing thoughts of suicide, including among those in a suicidal crisis. Although the number of studies included in these reviews is small, the evidence also points toward the potential utility of testimonies from others with similar experiences as a helpful suicide prevention strategy. In the United States, NowMattersNow [17]—a website designed specifically for US residents—provides evidence-based resources including videos, blogs, and suicide prevention strategies (eg, emotion self-regulation coping strategies) to promote the self-management of suicidal thoughts. The website is available 24 hours per day and is freely accessible to the public. Visitors to this website were found to stay on the website for an average of 1 minute 31 seconds, with a bounce rate (percentage of visitors who left after viewing 1 page) of 61% [18]. To put this in context, a bounce rate of 60%-90% is average for landing pages. Based on an embedded anonymous web user survey, Whiteside et al [18] found that engagement with the NowMattersNow website was associated with a self-reported reduction in feelings of suicidal ideation and distress. Given the cultural similarities between the United States and Scotland and the perceived demand of lived experience support from people who are suicidal [6,19], such a resource may be effective within the Scottish context.

### Aims

This pilot phase usability study is part of a wider, ongoing digital project to reduce suicide across Scotland. The website was co-designed and delivered by individuals with lived experiences of suicidal thoughts and behaviors. The overall aims of the project are listed in Multimedia Appendix 1. To help guide these aims, theory-driven outcomes for this study were determined in advance of website development using a logic model (Multimedia Appendix 2). Given the early stage of the development of this suicide prevention website, this study aimed to address the following short-term intended outcomes of the logic model. Specifically, the development of a suicide prevention website aimed to (1) support the Scottish population when experiencing a suicidal crisis that is codeveloped with the target population, (2) investigate which users of the website feel that they have received a compassionate response, (3) investigate which users experience a reduction in feelings of entrapment during engagement with the website, (4) investigate which users experience a reduction in suicidal thoughts during engagement with the website, and (5) investigate which users of the website find it accessible and relatable.

## Methods

### Study Design

The website, Surviving Suicidal Thoughts (SST) [20], was developed using an iterative approach involving a multidisciplinary website development team (comprised of digital experts, health professionals, and academic researchers) and direct feedback from the Scottish government’s Living and Lived Experience Panel (n=5; Scottish residents with lived experience of suicidal thoughts and behaviors).

### Ethical Considerations

Research permission was granted by the local National Health Service (NHS) Research and Development Committee in 2022. A Data Protection Impact Assessment was completed and approved prior to the launch of the web user survey. However, following NHS guidance, ethics approval is not required for service development and evaluation studies such as this (section 3.1 of the UK Policy Framework for Health and Social Care Research) [21]. Informed consent to participating in the anonymized feedback survey was inferred through participants opting in to the survey following the survey invitation appearing on the web page, and a privacy notice was available on the invitation page of the survey. Anonymous web user metrics and engagement with the website were collected only from users who had consented to allow tracking cookies on their internet-enabled device prior to “landing” on any NHS24 web page. Due to the anonymity of participation, participants could not be compensated.

### Data Collection

#### Overview

Data collection for this pilot phase spanned 41 weeks (see Multimedia Appendix 3 for the timeline). Originally, the timeline was expected to span 6 months; however, due to low participant engagement with the web user survey, data collection was extended to include the second marketing campaign spanning from October 2022 to February 2023. Visitors’ engagement with the website was monitored during development with adjustments made based on user behavior (Multimedia Appendix 3). Such behavior was monitored before and during visiting the website, which allowed SST visitors to be categorized into 1 of 5 user groups (defined in Table 1). The way in which the 5 visitor groups were expected to navigate to, and use, the SST website is depicted in Multimedia Appendix 4. Further details of the development and evaluation of the website are summarized under the following subheadings: “Website Content,” “Web User Evaluation Survey,” and “Marketing.”

**Table 1:** Surviving Suicidal Thoughts website visitor groups^a^.

User type	Behavior	Search term examples
1. Web-based suicide methods seeking	Individuals not looking for support for their suicidal thoughts	“Want to die” “End my life”
2. Web-based support seeker	Individuals actively looking for support for their suicidal thoughts	“Suicide support”
3. Told friends or family	Individuals who are looking for support for a friend or relative who they know is experiencing suicidal thoughts	“Suicidal relative,” “Suicidal friend”
4. Suspecting friends or family	Individuals who are looking for support for a friend or relative who they suspect may be experiencing suicidal thoughts; however, this has not been confirmed	“Is my friend suicidal?”
5. Generic curious or professional	Individuals not help-seeking for themselves or others and not in suicidal crisis	“Suicide prevention day”

^a^The 5 user groups identified to visit the Surviving Suicidal Thoughts website between June 2022 and February 2023 based on search terms entered into Google by Scottish residents.

#### Website Content

**Figure 1:**
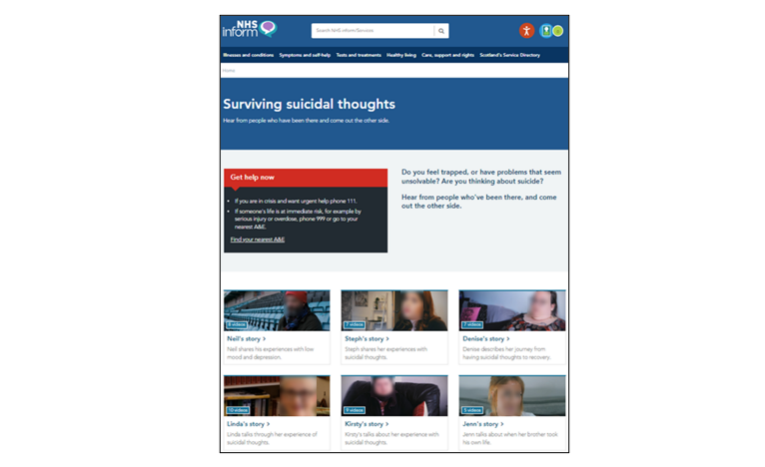
Image of the Surviving Suicidal Thoughts landing page.

SST was hosted on the NHS Inform internet platform, Scotland’s national health care website (Figure 1). The website included personal testimony videos of 5 people (Neil, Steph, Denise, Linda, and Kirsty) with lived experience of suicide and 1 person bereaved by suicide (Jenn). These videos were developed specifically for the website. In these videos, the speakers shared their broad experiences of low mood and suicidal thoughts and behaviors, rather than discussing specific details related to their exact experiences of suicidal crisis. Each video was cut into between 5 and 10 chapters, with each chapter being approximately 90 seconds each. The chapters were titled, which allowed visitors to jump between individual sections. Early in the first marketing campaign, it was observed that visitors of the website only explored the top 2 videos that appeared on their computer or phone screen. In response, the videos were rotated each month to minimize any order effects.

The website also featured emergency service contact information, signposting to third-sector mental health organizations, and how to respond to someone who discloses or may be experiencing suicidal thoughts. To directly evaluate the experiences of web users, the website included a pop-up survey modeled on NowMattersNow [17]. Engagement with the website was captured by the intervention delivery team at NHS Inform using Google Analytics [22].

#### Web User Evaluation Survey

The SST survey was developed using the HotJar survey platform [23]. Initial survey content and methodology were collated by HM (study author) and presented to a focus group of individuals with lived experience of suicidal thoughts and behaviors for their feedback (see Multimedia Appendix 5 for focus group feedback). To minimize overburdening SST website visitors, the Lived Experience Panel recommended reducing the survey items from 12 to 4 questions. As a result, it was intended that a series of “blocks” of survey items would be displayed during the pilot phase to capture information on a range of demographic and psychological factors over time. However, due to the limited engagement with the survey (see Overview in the Results section), it was decided that only the first block of questions would be used in the pilot phase to improve statistical power for analysis. The final survey items and measurement scales included in the pilot are presented in Multimedia Appendix 6; however, in brief: 2 items assessed participants’ intensity of suicidal ideation (taken from NowMattersNow [17]; range: 1=not at all and 5=completely overwhelming), 1 demographic variable explored participants’ age group (10-year increments from “younger than 18 years” to “70+ years”), and 1 item captured geographic location (based on NHS health board). A fifth item (reason for visiting the website) was added midway through survey recruitment. All survey items were asked at a single time point. The suicidal ideation items asked web users to recall the level of intensity of their suicidal thoughts at the time of first landing on the SST website as well as the current intensity of their suicidal ideation at the time of participating in the survey. These survey items were anonymous, and participants could discontinue the survey and return to the SST website at any time.

To be consistent with the format of NowMattersNow [17], the SST survey invitation initially appeared 3 minutes after landing on the SST website. However, after 2 days, it became evident that the average web user duration was less than 3 minutes (with only 4 visitors seeing the invitation); therefore, we adjusted the survey invitation to appear after 90 seconds (Multimedia Appendix 3) to match 50% of user traffic.

#### Marketing

Digital marketing was used to promote the website resources on social media platforms (ie, Meta [Facebook and Instagram], X [formerly known as Twitter], and Spotify). Live targeting platforms (Nano and Google Ads) were used to advertise the website to relevant web users. Marketing data were collected from social media platforms, from Nano and Spotify by a third party (Republic of Media) using a pay-per-click marketing campaign. Advertisements promoting the website were designed to convey empathy and hope. Following some prosuicide comments from the public, advertisements for the SST website on Meta platforms were discontinued 1 week after launch to protect the public. This funding was reallocated to Google Ads. Additionally, advertising on X was discontinued after 23 weeks (November 27, 2022) of this 41-week pilot phase. This was due to changes in X’s ownership and uncertainty around its operating procedures. Thereafter, funding for the promotion of the SST website on X was reallocated to Nano.

### Data Analysis

#### Website Content Engagement

Analysis was modeled on those used by Whiteside et al [18]. Website engagement was inferred using bounce rates (the percentage of web visitors who leave or “bounce” from a web page without taking follow-up action). Web users’ device used, date, time, and journey to the website were also captured. Video engagement (which videos and duration) was provided by Nano.

#### Web User Evaluation Survey

Data relating to the date, time, and type of internet-enabled device (eg, laptop and mobile phone) were summarized using frequencies and percentages collated by HotJar survey systems. Using G*Power [24], a priori power analysis determined that a minimum of 102 participants were needed for pairwise comparisons between baseline and follow-up data (effect size=0.03; α=.05; power=0.9). All statistical analyses were conducted using SPSS (version 28; IBM Corp). The distribution of the data was inspected visually and then tested using Kolmogorov-Smirnov tests, which indicated that the data were not normally distributed (*P*<0.001). Subsequently, a nonparametric approach was deemed appropriate to analyze the survey responses, thereby avoiding a type I error. Subsequently, a Wilcoxon 1-sampled, 2-tailed *t* test was used to statistically test the change in participants’ intensity of suicidal ideation. A statistical subanalysis of other outcomes (eg, age and geographic location) was not possible due to the small group sizes. Participants who did not answer both suicidal ideation questions were not included in the statistical analysis; all remaining missing data were grouped with “prefer not to say.”

#### Marketing

Information captured included engagement with advertisements (click-through rates [CTRs]) and videos (view-through rates) as well as demographic information (sex: male, female, or unknown and age group: 18-24, 25-34, 35-44, 45-54, 55-64, 65+ years, “unknown”). These data were reported in relation to advertising trends commonly observed in similar advertising strategies [25,26]. Additionally, Nano provided geographical information regarding advertising exposure and engagement based on the number of people who saw an advertisement (impressions).

## Results

### Overview

SST [20] was developed using an iterative approach between the web page development team and Scotland’s Living and Lived Experience Panel and remained live throughout the 41-week pilot phase (from June 28, 2022, to February 20, 2023). The SST website received 25,784 unique visitors equating to an average of 99.9 page views per 24-hour day. Daily traffic to the website peaked at 10 PM, with the greatest amount of traffic on Sundays.

Most visitors accessed the website via mobile phone (n=21,402, 83%), followed by desktop computer (n=3068, 11.9%). When compared to commercial metrics [22], social media marketing CTRs for SST were considered “good” on all platforms. For videos, the view-through rate was 80.2% (n=892,943), which was considered “high.” Journeys of SST users were most commonly via Google Search (n=6314, 24.3%), followed by Meta (n=4705, 17.1%). For more details of marketing expenses, marketing strategy, and user engagement per platform, please see Multimedia Appendix 7.

More “web-based suicide methods seeking” than “web-based support seeker” user journey members clicked on testimonial advertisements (advertisements that included names) or engaged with advertisements titled “hear from one whose been there.” However, sample sizes were too small to explore this statistically.

**Figure 2:**
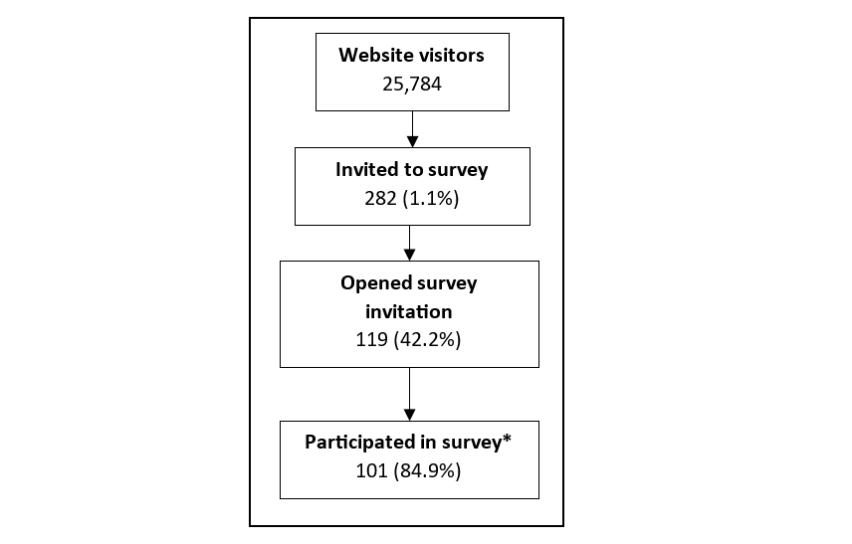
Participant flowchart of participant engagement with the Surviving Suicidal Thoughts website and evaluation survey between July 2022 and February 2023. *Answered at least 1 survey question.

The bounce rate to the website was 76.9%, suggesting that almost a quarter of website visitors clicked on a subpage of the SST website (eg, a video link). Of the web users who were invited to participate in the evaluation (n=282, 1.1%), 42.2% (n=119) opened the survey link. In total, 84.9% of those who opened the survey answered at least 1 question (Figure 2).

### Demographic Characteristics

Google Analytics indicated that 56% (n=14,439) of SST visitors were female, and most “web-based nonsupport seeking” groups were also indicated to be female. Web users who accessed the website through Google Ads were most likely to be aged between 25 and 34 years. In contrast, half (n=47, 49.6%) of the respondents to the evaluation survey were between 40 and 59 years (Table 2). Most (n=69, 66.4%) survey participants chose not to indicate which health board they lived in.

**Table 2:** Demographic and geographic summary of participant characteristics of the survey evaluation.

Variable					Values, n (%)
**Age (years)**					
	<18			6 (5)	
	19-29			11 (9.2)	
	30-39			11 (11.8)	
	40-49			14 (27.7)	
	50-59			33 (21.9)	
	60-69			26 (6.7)	
	70+			8 (0.8)	
	Prefer not to say or not applicable			7 (5.9)	
	Unanswered			13 (10.9)	
**Health board**					
	Ayrshire and Arran			5 (4.2)	
	Borders			1 (0.8)	
	Fife			2 (1.7)	
	Fourth Valley			2 (1.7)	
	Grampian			3 (2.5)	
	Greater Glasgow and Clyde			7 (5.9)	
	Highland			1 (0.8)	
	Lanarkshire			5 (4.2)	
	Lothian			9 (7.6)	
	Shetland			1 (0.8)	
	Tayside			3 (2.5)	
	Western Isles			1 (0.8)	
	Prefer not to say or not applicable			10 (8.4)	
	Unanswered			69 (58)	
**Reason for visit^a^**					
	I am looking for resources to help me cope if I feel suicidal again			9 (7.6)	
	I am thinking about suicide			14 (11.8)	
	I do not think I can keep myself safe			4 (3.4)	
	I have a plan for suicide			6 (5)	
	I am looking for resources to support someone else			8 (6.7)	
	Other or prefer not to say			11 (9.2)	
	Unanswered			67 (56.3)	
**Suicide intensity**					
	**Intensity of suicidal thoughts upon first visiting the site (mean 3.06, SD 1.58)**				
		1=not at all	30 (25.2)		
		2	12 (10.1)		
		3	19 (16)		
		4	19 (16)		
		5=completely overwhelming	30 (25.2)		
		Unanswered	9 (7.6)		
	**Intensity of suicidal thoughts at time of survey (“now”; mean 2.87, SD 1.59)**				
		1=not at all	33 (27.7)		
		2	11 (9.2)		
		3	17 (14.3)		
		4	16 (13.5)		
		5=completely overwhelming	24 (20.2)		
		Unanswered	18 (15.1)		

^a^Item added 14 weeks into data collection phase.

### Video Engagement

The first 2 videos that appear on a web visitor’s browser commonly received the most visitor engagement. “Neil’s story: Experiences with low mood and depression” and “Steph’s story: Experiences with suicidal thoughts” were the most viewed chapters. On average, web users watched 86.64% (SD 5.91%) of the video chapters. Chapters most likely to be viewed in full were titled “The anxiety underneath was so much to bear” (Kirsty, chapter 3) and “There is light at the end of the tunnel” (Neil, chapter 4).

### Suicidal Thoughts

Most (n=5290, 83.8%) CTRs via Google Ads were attributed to the “web-based support seeker group,” “told friends or family,” or “suspecting friends or family” SST visitor journey groups (Table 3). In contrast, 86.5% of CTRs from Google Search terms related to concern for others experiencing suicidal thoughts (50% CTR for “suicidal relative” and 36.5% CTR for “suicidal friend”), which represent the “told friends or family” and “suspecting friends or family” user journey groups (Table 1). However, 65.5% (n=67) of respondents to the evaluation survey did not indicate their reason for visiting the website (9.2% responded “other or prefer not to say” and 56.3% of participants did not answer).

**Table 3:** Total number of impressions and clicks to the Surviving Suicidal Thoughts website via adverts on social media and Google.

		Impressions, n	Clicks to website, n	CTR^a^ (%)
**SST user journey group**				
	Web-based suicide methods seeking^b^	17,282	920	5.32
	Web-based support seeker^c^	27,638	2898	10.49
	Told friends or family^d^	14,565	2172	14.91
	Suspecting friends or family^e^	1730	220	12.72
	Generic curious or professional^f^	1496	104	6.95
**Media platform**				
	X	1,209,432	12,847^g^	1.06
	Nano	1,124,972	3471	0.31
	Spotify	326,359	—^h^	0.26
	Facebook	202,326	4705	2.33
	Total	2,925,800	22,637	N/A^i^

^a^CTR: click-through rate.

^b^Individuals not looking for support for their suicidal thoughts.

^c^Individuals actively looking for support to relieve their suicidal thoughts.

^d^Friend or relative who they know to be experiencing suicidal thoughts.

^e^Friend or relative who they suspect may be experiencing suicidal thoughts.

^f^Those looking for general information on suicide prevention who does not otherwise fit in the other web user groups.

^g^Based on engagement with social media post, not clicks to website.

^h^Data not available as these were audio advertisements; therefore, clicking to the website was not possible.

^i^N/A: not applicable.

Based on survey responses, 25.2% (n=30) of visitors retrospectively described the intensity of their suicidal thoughts when first visiting the website as “completely overwhelming” compared to 20.1% (n=24) of participants at the time of survey engagement (Table 2). Overall, there was a significant reduction in the intensity of suicidal thoughts from the recollection of intensity when first landing on the web page to participating in the survey (*t*_100_=–1.89; *P*=.03). No statistically significant differences were observed between the intensity of suicidal thoughts as a function of age (≤49 years vs ≥50 years).

## Discussion

This study aimed to evaluate a pilot web-based self-help resource to help support residents of Scotland who have suicidal ideation. The findings of this study are discussed below in relation to the 5 short-term intended outcomes of the logic model (Multimedia Appendix 2).

### Principal Findings

The design and content of SST [20] was developed through consultation with the Living and Lived Experience Panel in Scotland. This input included direct contributions to the website videos—the defining feature of the SST website. Together with other stakeholders (digital specialists and health professionals), this led to a suicide prevention website launched in June 2022 specifically designed to support residents of Scotland. Therefore, outcome 1 (support the Scottish population when experiencing a suicidal crisis that is codeveloped with the target population) was achieved.

Due to low survey engagement, the survey blocks were not rotated as initially intended. Therefore, this pilot phase was unable to obtain visitor views on receiving a compassionate response from the SST website (eg, helpful videos and guidance). Despite this, amendments to the website and survey user evaluation (Multimedia Appendix 3) were conducted in response to user engagement. Thus, a compassionate approach was exhibited by the website developer team to meet the changing needs of the website visitors. Additionally, involvement from the Lived Experience Panel was intended to best serve the needs of the Scottish population. Their input included guidance on sensitive advertising of the website to best avoid triggering people who were not in distress. Additionally, on Meta, comments encouraging suicide prompted the team to discontinue the marketing campaign on this platform for the well-being of social media users. Subsequently, outcome 2 (users of the website feel that they have received a compassionate response) was only partially addressed.

Due to low survey engagement, questions about entrapment were excluded from the web user survey; therefore, outcome 3 (users experience a reduction in feelings of entrapment during engagement with the website) was not addressed. Despite this, the Lived Experience Panel expressed that both internal and external entrapment survey questions were acceptable items to be included within the SST website survey.

The survey data indicated that the intensity of suicidal thoughts had significantly reduced between the time participants first arrived on the SST website and the time they engaged with the survey. However, as the *P* value just met the traditional level of significance, this needs to be replicated in a large sample. Nonetheless, outcome 4 (users experience a reduction in suicidal thoughts during engagement with the website) was achieved.

Data from Google Analytics indicated that the website was accessed by all geographic regions of Scotland and people up to 70 years of age. This geographic spread of engagement was further reflected by data collected by Nano. Additionally, the website was used at all times of day and night and from a range of devices (eg, computers, phones, and tablets). These findings suggest that the website was both accessible and relatable to residents of Scotland; therefore, outcome 5 (users of the website find it accessible and relatable) was achieved.

### Comparison With Prior Work

Consistent with Lai et al [15] and Robinson et al [16], engagement with the SST [20] seemed to significantly reduce the intensity of suicidal thoughts of visitors to the website. The findings of this pilot study are similar to those of the NowMattersNow evaluation [18]. Specifically, the prevalence of web users who described the intensity of their suicidal thoughts as “completely overwhelming” when first visiting the site was similar across the 2 websites (25% of SST web users compared to 28% of NowMattersNow) [18]. Equally, both websites’ bounce rate was within the expected range according to web-based marketing norms of 60%-90% [26] (NowMattersNow [17]: 61% and SST [20]: 76.91%). Both websites reported an absence of participants aged 70+ years. Conversely, Scotland’s SST website was indicated to engage younger people (<18 years: 10.15% of SST visitors), whereas this age group was not captured by NowMattersNow [17].

The pattern of engagement with the SST websites is consistent with leading theories of learned behavior such as social learning theory [11]. Social learning theory posits that people are more likely to model their own behavior on those with whom they identify. Williams et al [19], based on a cross-sectional sample of self-harm web-based communities, found that such groups are more likely to seek informal help from perceived peers than professional services, and advice on self-care was reportedly more effective when provided by these peer supports. Social identification was evident in this study, for example, where web users were more likely to engage with advertisements or SST videos, which corresponded with their own age or sex.

Most visitors were aged between 25 and 34 years, consistent with “Steph,” whose video was viewed the most compared to all other videos on the SST website. Additionally, most visitors accessing the SST website via “X” (formerly known as Twitter) were typically male participants and landed on the SST website via advertisements about “Neil’s story.” Similarly, the marketing company used in this study (Republic of Media) reported that, in general, female participants were more likely than male participants to engage with Google Ads. In this study, advertisements relating to “Linda’s story” were most likely to be engaged with on Google platforms. Although these findings are preliminary, these trends would suggest that it may be beneficial to consider principles of social learning theory [11] when developing suicide prevention websites.

This pilot phase usability study was developed to inform a larger study aimed to reduce suicide in Scotland via timely peer-led support. Subsequently, recommendations based on the findings here include (1) updating marketing strategies to meet the needs of those seeking and not seeking help for their suicidal thoughts, (2) using pop-up invitations for the user evaluation to improve survey engagement, and (3) increasing the demographic diversity of populations represented in the survivor videos. As illustrated by the logic model (Multimedia Appendix 2), the long-term goals aim to integrate the website into the health care landscape, which clinical and nonclinical populations can use as an established resource to support well-being. The current evaluation also indicates that future work should consider the role of the learned behavior (eg, social learning theory) [11] in relation to engagement with suicide prevention resources. Additionally, to explore the extent to which web visitors felt that they received compassionate support, qualitative interviews with individuals who have used the website would be beneficial.

### Limitations

The study findings need to be interpreted in the context of the limitations related to the content of the website, the web user survey, and how the website was marketed. First, most web users usually only engaged with the first 2 videos that appeared on their browser; however, this trend was only identified after the website was live. Although Neil’s story and Steph’s story were the most engaged with by web users, this may have reflected their position on the website, as opposed to the web users’ video preference overall. Additionally, it is unknown whether engagement with the website provided a compassionate response to its visitors.

Second, due to constraints of the survey platform used in this intervention, not all preferred approaches could be implemented into the survey methods. Namely, the survey could only appear once to web users, thereby limiting the survey to a cross-sectional design, where the intensity of suicidal ideation was explored both retrospectively and currently. Additionally, some of the recommendations made by the Lived Experience Panel could not be incorporated into the survey (eg, having the survey invitation being a permanent feature on the screen so website visitors could participate in the survey whenever they felt ready). This may account for the low engagement with the SST survey. Additionally, although the study originally followed the Lived Experience Panel’s recommendation of a 3-minute delay before the survey invitation pop-up, due to the average duration of visits to the SST website being shorter than anticipated, the invitation was reduced to 90 seconds, consistent with NowMattersNow [18]. Although survey participation rates were good, the number of people who saw the invitation was lower than expected, limited to those who stayed on the website long enough to see the invitation, and engaged by those who chose to participate in the survey. Subsequently, the findings are unlikely to be generalizable to the wider population of Scotland. The low number of survey invitations was likely due to web users having disabled cookies across the NHS platform. This lower recruitment subsequently led to the blocks of survey items not being rotated; therefore, additional information (eg, sex) not being captured in the data. The small participant sample also limited subgroup analyses to age comparisons only as well as preventing further survey items (eg, entrapment) from being substituted into the survey over time. Given that most participants did not answer the self-reported question relating to the health board, it is unclear whether the participant sample was reflective of the overall SST web user population. Additionally, the final survey item included responses for individuals who were help-seeking for themselves, while those who were seeking help for others were grouped together with those who responded with “prefer not to say.” Therefore, it was not possible to make comparisons between reasons for visits in relation to demographic characteristics or suicidal distress. Finally, most respondents to the survey were aged between 40 and 59 years, whereas Google Analytics indicated that most web users were aged between 25 and 34 years. This indicates that pop-up surveys may not be the most effective way to capture web user satisfaction on self-help platforms such as SST and that the findings from the survey may not be generalizable to all visitors to the website.

Third, the marketing approach of the website must be considered. Word limits on Google Ads may have impacted on the success of engaging certain demographics, namely male participants. This is reflected in the alternative advertising platforms, such as Spotify, where proportionately, non–help-seeking male participants were more likely to engage with SST advertisements than non–help-seeking female participants. Challenges were encountered with advertising, with advertising being discontinued on some platforms. This highlights a challenge between balancing a soft-marketing strategy against the need for ethical considerations such as safeguarding to ensure public well-being. Solutions to achieving this balance may include the use of trigger warnings and further developments in social media algorithms.

Finally, the bounce rate may not have provided a true representation of the SST websites’ utility by the user. Specifically, the bounce rate of the SST was higher than NowMattersNow [18], reflective of web users leaving the website after arriving on the landing page. However, it is unclear whether the bounce rate was due to visitors benefiting from the website approach to making information salient (eg, crisis card information on the landing page) or because the website did not meet the individual’s expectations during their visit, thereby prompting them to leave. Therefore, the bounce rate (ie, engagement) may not necessarily be reflective of the website failing to meet the need, but that the landing page provided sufficient information for the web user.

### Conclusions

A codeveloped website for suicide prevention, designed for the Scottish population, may help to reduce the intensity of one’s suicidal thoughts. Marketing of the website across various social media, entertainment, and internet search platforms is feasible. The website was particularly popular with those who were looking for help for themselves, and principles of social learning theory may play a role in optimizing the utility of such resources. A larger study is required to explore the effectiveness of such a website and determine whether it works for different subgroups of the Scottish population.

## Appendix

Multimedia Appendix 1 Project aims of the Surviving Suicidal Thoughts website.

Multimedia Appendix 2 Logic model summarizing the anticipated inputs, outputs, and outcomes of the Surviving Suicidal Thoughts website.

Multimedia Appendix 3 Timeline illustrating developmental milestones of the Surviving Suicidal Thoughts website between 2020 and 2023.

Multimedia Appendix 4 The 5 user journeys anticipated visitors to the Surviving Suicidal Thoughts (SST) website will take from using a search engine to landing on the SST website.

Multimedia Appendix 5 Collated feedback form a Lived Experience Panel in February 2022 regarding the potential survey items to be included in the Surviving Suicidal Thoughts website.

Multimedia Appendix 6 Survey items included in the Surviving Suicidal Thought website cross-sectional user evaluation survey from July 2022 to February 2023.

Multimedia Appendix 7 Summary of social media costs for the Surviving Suicidal Thoughts website first marketing campaign from July to September 2022.

## Abbreviations

CTR: click-through rate
NHS: National Health Service
SST: Surviving Suicidal Thoughts

## Data Availability

The datasets generated and analyzed during this study are available from the corresponding author on reasonable request.
