# Supplementary material for: Exploring Web-Based Support for Suicidal Ideation in the Scottish Population: Usability Study
Source: JMIR Form Res. 2025 Jan 24;9:e55932. doi: 10.2196/55932 (PMC11806263; doi:10.2196/55932)
Supplement: Multimedia Appendix 1 [file formative_v9i1e55932_app1.docx]

- To create a sustainable online resource to promote suicide reduction within the Scottish population
- Develop an evidence-based, online resource (co-developed between those with lived experience of suicidal behaviour and those with theoretical and/ or clinical knowledge of suicidal behaviour) is assimilated into the existing suicide prevention landscape, integrated within existing national clinically governed structures.
- Gain considerable reach and promotion of the resource via online platforms, therein engaging populations indicated to be most vulnerable to suicidal ideation and behaviour, as well as those in remote geographical locations
- Include an embedded evaluation to assess the feasibility of the suicide prevention resource
