# Supplementary material for: Exploring Web-Based Support for Suicidal Ideation in the Scottish Population: Usability Study
Source: JMIR Form Res. 2025 Jan 24;9:e55932. doi: 10.2196/55932 (PMC11806263; doi:10.2196/55932)
Supplement: Multimedia Appendix 2 [file formative_v9i1e55932_app2.docx]

Appendix 2. Logic model summarizing the anticipated inputs, outputs, and outcomes of the Surviving Suicidal Thoughts website

| ***Inputs*** |  | ***Outputs***  *Activities Participation* | |  | ***Outcomes*** *(Impact)*  *Short Medium Long* | | |
| --- | --- | --- | --- | --- | --- | --- | --- |
| *People***:** Individuals with lived experience (peers), clinical & non-clinical professionals, research academics, project  *Finances***:** Funding by the Chief Scientist Office  *Equipment*; Laptops/ computers, Microsoft packages, video conferencing software, specialist data analysis software (SPSS, Comprehensive Meta-Analysis  *Existing research:* Actions 5 and 6 Stakeholder Feedback Survey Report (September 2020) |  | *Website*: provision of videos of lived experiences, signposting to support services.  *Advertising*: Promotion of peer support content, and immediate support.  *Data collection*:  i) Qualitative interviews with website users.  ii) Quantitative data capture of utility of website and engagement with follow-up services. | Use of website.  Engagement with occasional surveys to assess suicidality.  Completion of semi-structured interviews.  Data collection of service engagement.  Engagement from those experiencing a suicidal crisis or have been affected by others’ suicidal behaviour. |  | (IO1) Development of peer-led suicide prevention website to support the Scottish population when experiencing a suicidal crisis.  (IO2) Users of the website feel that they have received a compassionate response.  (IO3) Users experience a reduction in feelings of entrapment during engagement with the website.  (IO4) Users experience a reduction in suicidal thoughts during engagement with the website.  (IO5) Users of the website find it accessible and relatable. | (IO6) Improved signposting for individuals experiencing a suicidal crisis.  (IO7) Clinical and non-clinical professionals signpost people in distress to the website.  (IO8) Adults from a wide range of backgrounds (age, gender geography, and ethnicity) use the website. | (IO9) Reduction in suicidal crisis, self-harm and suicide in Scotland.  (IO10) Reduced burden on emergency services (including hospital-based and call-out services) for suicidal crisis support.  (IO11) Website is an accepted resource to be used in suicide prevention in Scotland. |

| Assumptions  Website users are experiencing a suicidal crisis or seeking help for a loved one experiencing a suicidal crisis.  Individuals experiencing a suicidal crisis will engage with internet search engines.  Individuals experiencing a suicidal crisis are aware of the website or can recognise its potential when browsing the internet. |  | External Factors  People in suicidal crisis may use a range of different suicide prevention resources. It may therefore be difficult to disentangle the unique impact of the website.  The indirect effects of access to the website for information by loved ones may be difficult to assess. |
| --- | --- | --- |
