# Supplementary material for: Exploring Web-Based Support for Suicidal Ideation in the Scottish Population: Usability Study
Source: JMIR Form Res. 2025 Jan 24;9:e55932. doi: 10.2196/55932 (PMC11806263; doi:10.2196/55932)
Supplement: Multimedia Appendix 3 [file formative_v9i1e55932_app3.docx]

| Year | Date | Event |
| --- | --- | --- |
| **2020** | 10^th^ September | Stakeholder survey feedback highlights the need for a co-developed online resource. |
| **2021** | August | NHS Scotland approves the development of a suicide prevention website. |
| **2022** | 8^th^ February | Lived Experience Panel focus groups are conducted to gather feedback on the content of the SST website. |
|  | 9^th^ June | SST website is launched. |
|  | 24^th^ of June | 1^st^ marketing campaign commences (Google Ads, Facebook, Instagram, Twitter) |
|  | 28^th^ June | Advertising discontinued on Facebook and Instagram |
|  | 27^th^ June | Web user evaluation survey launched. |
|  | 29^th^ June | Timing of survey invitation reduced from 3 minutes to 90 seconds. |
|  | 11^th^ September | 1^st^ marketing campaign ends |
|  | 11^th^ October | Evaluation survey updated (see appendix 4). |
|  | 27^th^ October | 2^nd^ marketing campaign commences (Google Ads and Nano). |
|  | 5^th^ November | Goggle Ad Words and Nano marketing commences, |
|  | 2^nd^ December | Search term ‘prevent suicide’ was removed from the media campaign |
| **2023** | 20^th^ February | Survey data extracted for analysis. |
|  | 25^th^ March | 2^nd^ marketing campaign ends and marketing data is extracted for analysis. |
