# Supplementary figures and images for: Exploring Web-Based Support for Suicidal Ideation in the Scottish Population: Usability Study
Source: JMIR Form Res. 2025 Jan 24;9:e55932. doi: 10.2196/55932 (PMC11806263; doi:10.2196/55932)

Appendix 4 Surviving Suicidal Thoughts website user journeys


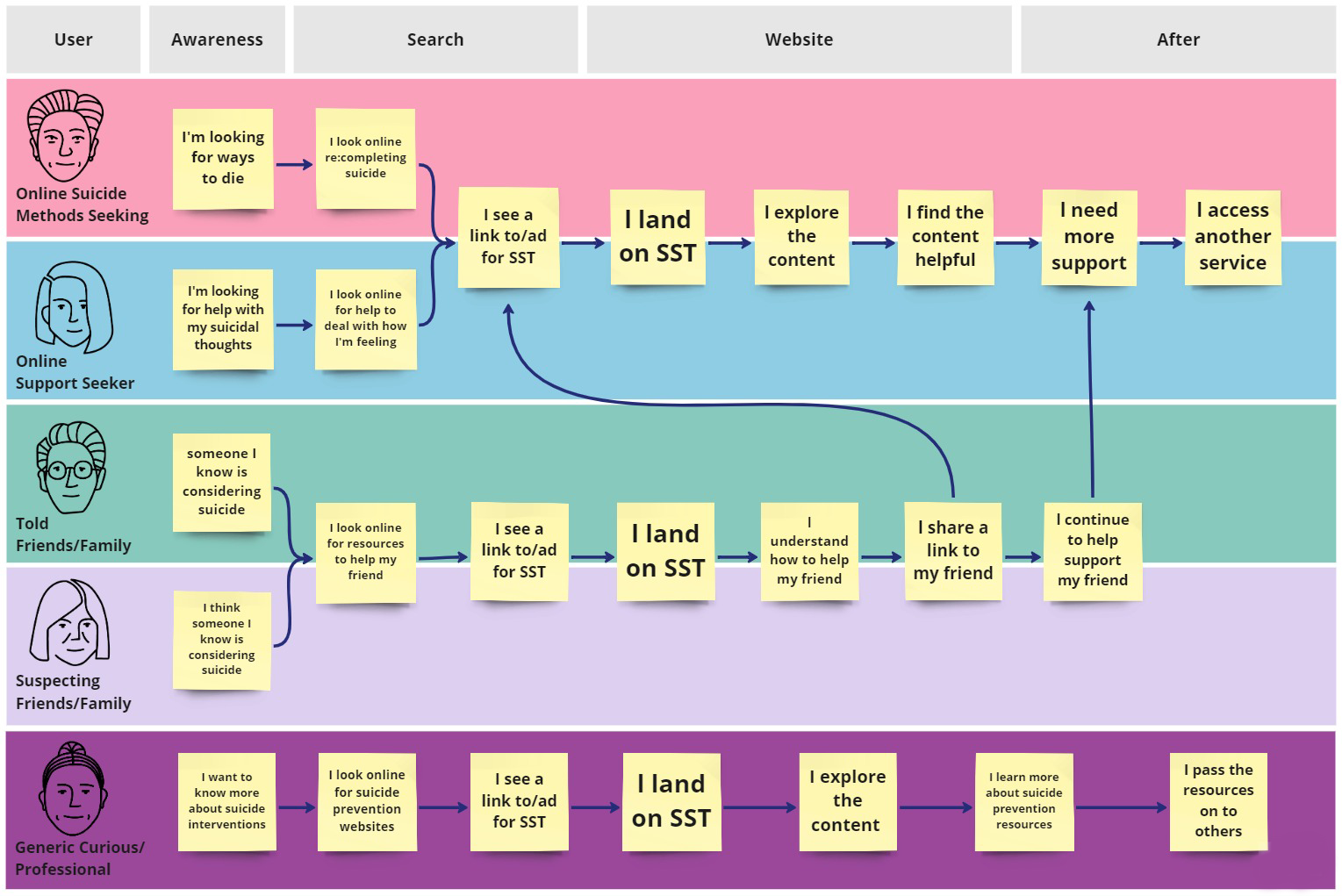

Supplement: Multimedia Appendix 4 [file formative_v9i1e55932_app4.docx]
