# Supplementary material for: Exploring Web-Based Support for Suicidal Ideation in the Scottish Population: Usability Study
Source: JMIR Form Res. 2025 Jan 24;9:e55932. doi: 10.2196/55932 (PMC11806263; doi:10.2196/55932)
Supplement: Multimedia Appendix 5 [file formative_v9i1e55932_app5.docx]

Appendix 5. Lived experience panel cross-sectional feedback (February 2022)

1. The survey would be limited to four items, which would be changed periodically to capture all the intended information
2. Approved survey items are:
   - Entrapment (responses from 0= ‘Not at all like me’, to 4= ‘Extremely like me’)
     - I feel powerless to change things
     - I often have the feeling that I would just like to run away
   - Distress: (responses from 0= ‘no distress’, to 10= ‘extreme distress’)
     - Please rate the intensity of your level of distress (including anxiety, depression, emotional pain etc.) when you first entered this site
     - What level is your distress now?
   - Suicidal ideation: (responses from 1= ‘Not at all overwhelming’ to 5= ‘Completely overwhelming’)
     - When you first entered this site, please rate the intensity of your suicidal thoughts.
     - What level are the intensity of your suicidal thoughts now?
   - Demographics:
     - What is your age range? (Under 18 years, 19-29 years, 30- 39 years, 40 – 49 years, 50 – 59 years, 60 – 69 years, 70+ years)
     - What is your gender? (Male, Female, Other)
     - Which region do you live in? (Greater Glasgow and Clyde, Lothian, Grampian, Highlands and Islands, Borders, Forth Valley, Lanarkshire, Tayside, Other)
     - What is the purpose of your visit?
3. Questions will be written using informal language
4. The survey invitation will be in pop-up format, appearing after three minutes, and will clearly state that the survey questions are designed to improve the website
5. The survey invitation will appear on the website after three minutes the individual landing on the website
6. Demographic information should be asked for last
