# Supplementary material for: Exploring Web-Based Support for Suicidal Ideation in the Scottish Population: Usability Study
Source: JMIR Form Res. 2025 Jan 24;9:e55932. doi: 10.2196/55932 (PMC11806263; doi:10.2196/55932)
Supplement: Multimedia Appendix 6 [file formative_v9i1e55932_app6.docx]

Appendix 6. Survey items placed on the Surviving Suicidal Thoughts website for the cross-sectional user evaluation from July 2023 to February 2023

Rate the intensity of your suicidal thoughts when you first entered the website:

- - 1 – not at all
  - 2
  - 3
  - 4
  - 5 – completely overwhelming
  - Prefer not to say/ not applicable

1. Rate the intensity of your suicidal thoughts now:
   - 1 – not at all
   - 2
   - 3
   - 4
   - 5 – completely overwhelming
   - Prefer not to say/ not applicable
2. Which health board do you live in?
   - Ayrshire and Arran
   - Borders
   - Fife
   - Forth Valley
   - Grampian
   - Greater Glasgow and Clyde
   - Highland
   - Lanarkshire
   - Lothian
   - Shetland
   - Tayside
   - Western Isles
   - Prefer not to say/ not applicable
3. Why are you visiting the website? (item added 26 weeks into data collection)
   - I am thinking about suicide
   - I have a plan for suicide
   - I don’t think I can keep myself safe
   - I am looking for resources to help me cope if I feel suicidal again’
   - Other/ prefer not to say
4. What is your range?
   - Under 18 years
   - 19-29 years
   - 30- 39 years
   - 40 – 49 years
   - 50 – 59 years
   - 60 – 69 years
   - 70+ years
   - Prefer not to say/ not applicable
