# Supplementary material for: Exploring Web-Based Support for Suicidal Ideation in the Scottish Population: Usability Study
Source: JMIR Form Res. 2025 Jan 24;9:e55932. doi: 10.2196/55932 (PMC11806263; doi:10.2196/55932)
Supplement: Multimedia Appendix 7 [file formative_v9i1e55932_app7.docx]

Appendix 7. Promotion, advert engagement and costs of marketing the Surviving Suicidal Thoughts website between June and September 2022

Twitter (24th June – 25^th^ September 2022)

- Spend: £4,729.63 to drive 1,209,432 impressions and 12,847 tweet engagements, including 8,147 clicks, 198 retweets, and 22 follows.
- The engagement rate remained consistently strong at 1.06% across the course of the campaign.
- The keyword 'therapy' drove the strongest engagements at 1.07%.

Nano (24th June – 8^th^ August 2022)

- The first burst of the Nano activity (24th June - 8th August), over delivered based on the plan, spending £8,133.85 to drive 191,466 impressions (vs. the 160,000 planned) and 162,677 video completions at an average view-through rate of 85%.

Spotify

- 1^st^ campaign (24th June - 10th July 2022):
  - Spent £2,000 to drive 200,315 impressions at a completion rate of 95.80% and a CTR of 0.36%.
  - The activity reached 52,287 unique users and drove a strong completion rate of 95.80%.
- 2^nd^ Campaign (22nd August - 11th September 2022):
  - Spend: £1,18.45 of the total budget, driving 126,044 impressions and at a completion rate of 97.67% and a distinctly weaker click-through rate of 0.15%.
  - Of musical genres, 20% of impressions were served to pop listeners, which is also where we saw the strongest click-through rate, Rock, and Hip Hop followed this.
  - Neil 4 drove the strongest click-through rate (0.18%), with Neil 5 driving the weakest, potentially due to the snippet being shorter & containing less information than other variations.

Meta (24^th^-28^th^ June 2022)

- Spend was £730.04 to drive 202,326 impressions and 4,705 link clicks at a very strong average click-through rate of 2.33%.
- Although traffic was the objective, the campaigns also drove 16,443 ThruPlays.
- The Ad ‘Kirsty 1’ drove particularly strong engagement, with 276 post reactions, 27 post comments,12 post saves and 27 post shares. Comments posted on these adverts indicate that the majority were users either empathising with the story or sharing their own.
